# Subtype-selective induction of apoptosis in translocation-related sarcoma cells induced by PUMA and BIM upon treatment with pan-PI3K inhibitors

**DOI:** 10.1038/s41419-023-05690-7

**Published:** 2023-02-27

**Authors:** Sho Isoyama, Naomi Tamaki, Yutaka Noguchi, Mutsumi Okamura, Yuki Yoshimatsu, Tadashi Kondo, Takeshi Suzuki, Shin-ichi Yaguchi, Shingo Dan

**Affiliations:** 1 Division of Molecular Pharmacology, Cancer Chemotherapy Center, Japanese Foundation for Cancer Research, 3-8-31 Ariake, Koto-ku, Tokyo, 135-8550 Japan; 2 Department of Patient-derived Cancer Model, Tochigi Cancer Center, 4-9-13 Yohnan, Utsunomiya, Tochigi, 320-0834 Japan; 3 Division of Rare Cancer Research, National Cancer Center Research Institute, 5-1-1 Tsukiji, Chuo-ku, Tokyo, 104-0045 Japan; 4 Division of Functional Genomics, Cancer Research Institute, Kanazawa University, Kakuma-machi, Kanazawa, Ishikawa, 920-1192 Japan; 5 OHARA Pharmaceutical Co., Ltd., 36F St. Luke’s Tower, 8-1 Akashi-cho, Chuo-ku, Tokyo, 104-6591 Japan

**Keywords:** Sarcoma, Targeted therapies

## Abstract

Translocation-related sarcomas (TRSs) harbor an oncogenic fusion gene generated by chromosome translocation and account for approximately one-third of all sarcomas; however, effective targeted therapies have yet to be established. We previously reported that a pan-phosphatidylinositol 3-kinase (PI3K) inhibitor, ZSTK474, was effective for the treatment of sarcomas in a phase I clinical trial. We also demonstrated the efficacy of ZSTK474 in a preclinical model, particularly in cell lines from synovial sarcoma (SS), Ewing’s sarcoma (ES) and alveolar rhabdomyosarcoma (ARMS), all of which harbor chromosomal translocations. ZSTK474 selectively induced apoptosis in all these sarcoma cell lines, although the precise mechanism underlying the induction of apoptosis remained unclear. In the present study, we aimed to determine the antitumor effect of PI3K inhibitors, particularly with regards to the induction of apoptosis, against various TRS subtypes using cell lines and patient-derived cells (PDCs). All of the cell lines derived from SS (six), ES (two) and ARMS (one) underwent apoptosis accompanied by the cleavage of poly-(ADP-ribose) polymerase (PARP) and the loss of mitochondrial membrane potential. We also observed apoptotic progression in PDCs from SS, ES and clear cell sarcoma (CCS). Transcriptional analyses revealed that PI3K inhibitors triggered the induction of PUMA and BIM and the knockdown of these genes by RNA interference efficiently suppressed apoptosis, suggesting their functional involvement in the progression of apoptosis. In contrast, TRS-derived cell lines/PDCs from alveolar soft part sarcoma (ASPS), CIC-DUX4 sarcoma and dermatofibrosarcoma protuberans failed to undergo apoptosis nor induce PUMA and BIM expression, as well as cell lines derived from non-TRSs and carcinomas. Thus, we conclude that PI3K inhibitors induce apoptosis in selective TRSs such as ES and SS *via* the induction of PUMA and BIM and the subsequent loss of mitochondrial membrane potential. This represents proof of concept for PI3K-targeted therapy, particularly such TRS patients.

## Introduction

Soft tissue sarcomas (STSs) are a rare and heterogeneous group of malignant tumors of mesenchymal origin featuring 100 different histological subtypes [1]. The outcome of patients with the early stages of STS have improved over the past few decades; however, the outcomes for advanced and non-resectable STS are unsatisfactory due to the lack of effective therapeutic drugs. The first-line treatment for advanced STS includes classical chemotherapeutic agents such as doxorubicin, ifosfamide and dacarbazine [2, 3]. However, only three new anticancer drugs (pazopanib, trabectedin and eribulin) have been approved in Japan since 2012 as second-line or subsequent treatment options for patients with advanced STS; furthermore, the efficacy of these drugs is limited [4]. Approximately 20% of STSs are classified as translocation-related sarcomas (TRSs) which harbor oncogenic fusion genes generated by chromosome translocation [5]. Fusion genes, such as *SS18-SSX*, *EWSR1-FLI1* and *PAX3-FOXO1*, are responsible for the generation of synovial sarcoma (SS), Ewing’s sarcoma (ES) and alveolar rhabdomyosarcoma (ARMS), respectively [5–10]. Most fusion gene products found in STSs serve as transcriptional factors or are involved in chromatin remodeling; however, unlike the activated kinases found in carcinoma, transcriptional factors are considered ‘undruggable’ because they lack catalytic active sites for drugs to bind [5]. However, effective targeted therapies for these TRSs have yet to be established.

Phosphatidylinositol 3-kinase (PI3K) is strongly activated in cancer cells by the activation of receptor tyrosine kinase, a gain-of-function hotspot mutation in the *PIK3CA* gene or the loss of phosphatase and tensin homolog (PTEN) expression, thus contributing to oncogenesis, proliferation and survival [11, 12]. Therefore, targeting PI3K is thought to be a promising therapeutic option for treating patients with a wide range of cancers featuring the activation of PI3K [13]. In fact, many PI3K inhibitors have been developed and investigated in clinical trials; however, results from clinical trials testing the use of PI3K inhibitors for solid tumors have been disappointing [14, 15]. The only exception was alpelisib, a PI3Kα-selective inhibitor that was approved by the U.S. Food and Drug Administration (FDA) for patients with *PIK3CA*-mutated HR^+^HER2^-^ breast cancer [16]. In contrast, the cell proliferation and survival of B-cell malignancies, such as chronic lymphocytic leukemia/small lymphocytic lymphoma (CLL/SLL) and follicular lymphoma (FL) have been shown to be dependent on PI3Kδ which plays a fundamental role in B-cell receptor-downstream signaling. PI3K inhibitors with potent inhibitory activity on PI3Kδ have been developed and approved for treating such B-cell malignancies [17]. The role of PI3K in the proliferation and survival of sarcoma is largely unknown; however, we previously reported that ZSTK474, a PI3K inhibitor, showed long-term disease stability in three out of four sarcoma patients enrolled in a Phase 1b clinical trial for cancer patients harboring solid tumors [18, 19]. These results prompted us to determine which subtypes of sarcoma respond to PI3K inhibitors. To this end, we established a sarcoma panel consisting of 14 sarcoma cell lines from various origins and found that the PI3K inhibitor exhibited significant antitumor activity and induced apoptosis in SS, ES and ARMS cell lines, all of which harbor chromosomal translocations [20]. However, the mechanism by which PI3K inhibitors induced apoptosis in these TRS subtypes and the effects of PI3K inhibitors against other TRS subtypes remain unclear.

In the present study, we aimed to determine the antitumor effects and the mechanisms of action, focusing particularly on apoptosis induction, of PI3K inhibitors using an expanded TRS panel consisting of cell lines established elsewhere and patient-derived cells (PDCs) originally established from resected tumors of various TRS origin. We found that PI3K inhibitors induced apoptosis selectively in SS, ES, ARMS and clear cell sarcoma (CCS) but induced only cytostatic effects and not apoptotic cell death in alveolar soft part sarcoma (ASPS), non-TRS or carcinoma cells. We also found that the induction of apoptosis was mediated by the selective induction of proapoptotic BCL-2 family members such as PUMA and BIM in cells derived from such TRS subtypes. Thus, we concluded that PI3K inhibitors selectively induce apoptosis in TRS such as SS, ES, ARMS and CCS due to the induction of PUMA and BIM expression. These results provide us with a proof-of-concept for PI3K-targeted therapy especially for patients with such TRS subtypes, with potential implications for future clinical applications.

## Results

### The subtype specificity of TRS undergoing apoptosis upon treatment with PI3K inhibitors

**Fig. 1:**
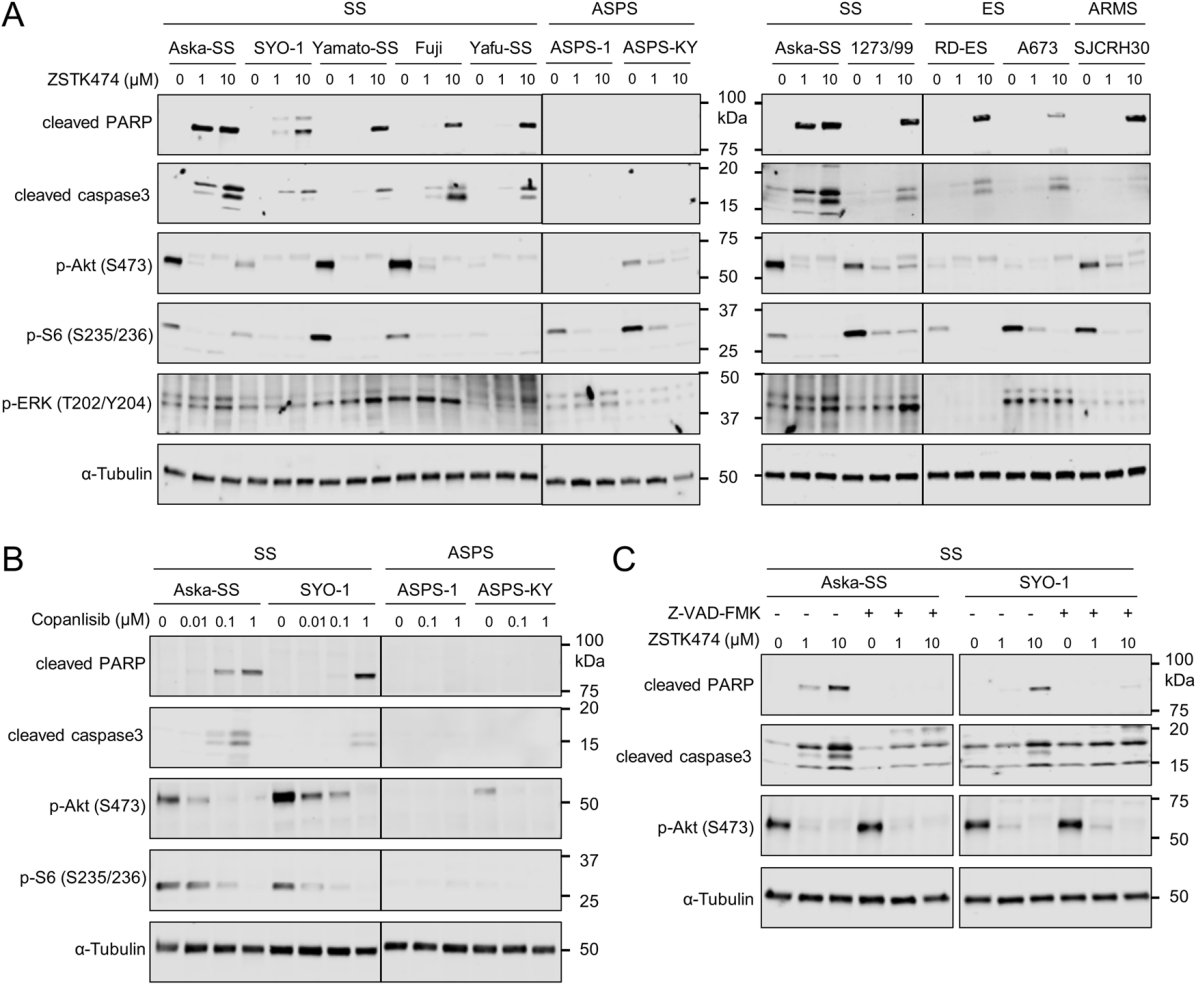
Treatment with PI3K inhibitors induced apoptosis in translocation-related sarcomas (TRSs) including synovial sarcoma (SS), Ewing’s sarcoma (ES) and alveolar rhabdomyosarcoma (ARMS) but not alveolar soft part sarcoma (ASPS). **A**, **B** Effects of the PI3K inhibitors ZSTK474 (**A**) and copanlisib (**B**) on PI3K signaling and apoptosis in the indicated TRS cell lines. TRS cells were treated with ZSTK474 or copanlisib at the indicated concentrations for 48 h. Lysed samples were immunoblotted to detect the phosphorylation of signal molecules including Akt (Ser473), S6 (Ser235/236) and ERK (T202/Y204) and the expression of apoptosis markers including cleavage of PARP and activation of caspase3 and α-Tubulin. **C** The effect of ZSTK474 on PI3K signaling and apoptosis in Aska-SS and SYO-1 upon treatment with Z-VAD-FMK. Aska-SS and SYO-1 cells treated with or without Z-VAD-FMK at 40 μM were treated with ZSTK474 at the indicated concentrations for 48 h. Lysed samples were immunoblotted to detect the phosphorylation of Akt (Ser473), cleavage of PARP, activation of caspase3 and expression of α-Tubulin. These experiments were performed independently at least two times with similar results.

First, we examined the effects of PI3K inhibitors on PI3K-downstream signals and apoptotic progression in cell lines derived from various TRS subtypes. ZSTK474 reduced the phosphorylation of PI3K signaling molecules including Akt (p-Akt) and ribosomal protein S6 (p-S6) in all cell lines tested, indicating the effective blockade of PI3K-downstream signaling. In contrast, ZSTK474 selectively induced the activation of caspase3 and the cleavage of poly-(ADP-ribose) polymerase (PARP) in SS, ES and ARMS but not ASPS cell lines (Fig. 1A). Similar results were obtained with copanlisib, another PI3K inhibitor (Fig. 1B). The progression of apoptosis was blocked upon pretreatment with Z-VAD-FMK, a pan-caspase inhibitor (Fig. 1C). In contrast, sarcoma cell lines without a known chromosomal translocation (non-TRS cell lines) did not undergo apoptosis after exposure to ZSTK474 for up to 48 h, as we previously demonstrated [20] (Fig. S1). These results indicated that the blockade of PI3K activities by ZSTK474 or copanlisib caused apoptotic caspase activation selectively in TRS cell lines derived from SS, ES and ARMS, but not in those derived from ASPS and non-TRS subtypes.

**Fig. 2:**
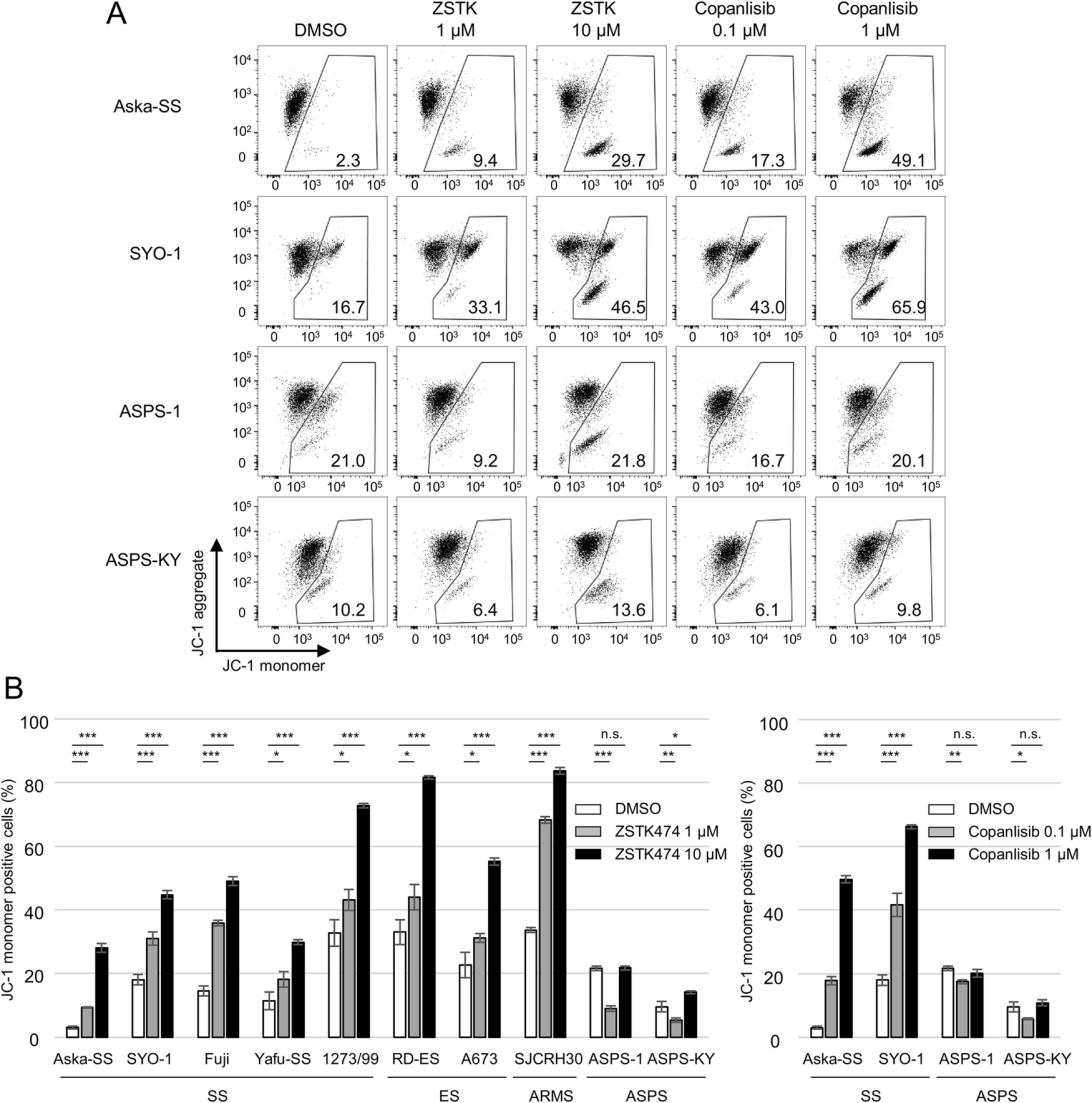
SS, ES and ARMS but not ASPS cell lines lost mitochondria membrane potential upon treatment with PI3K inhibitors. **A**, **B** Effects of the PI3K inhibitors ZSTK474 and copanlisib on mitochondrial membrane potential in TRS cell lines including SS, ES, ARMS and ASPS. TRS cells were treated with ZSTK474 or copanlisib at the indicated concentrations for 48 h. To examine the loss of mitochondria membrane potential, TRS cells were stained with JC-1 and analyzed by flowcytometry. JC-1 aggregates in healthy mitochondrial matrix; this can be visualized as red fluorescence. However, in dysfunctional mitochondria, JC-1 effluxes to the cytoplasm and exists as monomers with green fluorescence. Representative flow cytometric analysis (**A**) and summaries of triplicate data (**B**) are shown. The numbers in the panels (**A**) indicate the frequencies of JC-1 monomer positive cells. Data are means ± SD. These experiments were performed in triplicate and independently at least two times with similar results. Statistical analyses were performed by Dunnett’s test (**B**). *P < 0.05; **P < 0.01; ***P < 0.001.

It is well known that the increased permeability of the mitochondrial membrane causes the loss of membrane potential and the leakage of cytochrome c into the cytoplasm, thus leading to the subsequent activation of caspases to execute the apoptosis process [21]. Therefore, we examined the membrane potential of sarcoma cells upon treatment with PI3K inhibitors using JC-1, a mitochondrial membrane potential indicator [22]. Consistent with the activation of caspase3 and the cleavage of PARP, SS, ES and ARMS (but not ASPS cells) exhibited increased percentages of cells with a loss of mitochondria membrane potential in response to treatment with ZSTK474 or copanlisib (Fig. 2A and B). These results indicated that PI3K inhibitors induce apoptosis *via* increased permeability of the mitochondrial membrane.

### Transcriptome analysis of TRS cells treated with ZSTK474

**Fig. 3:**
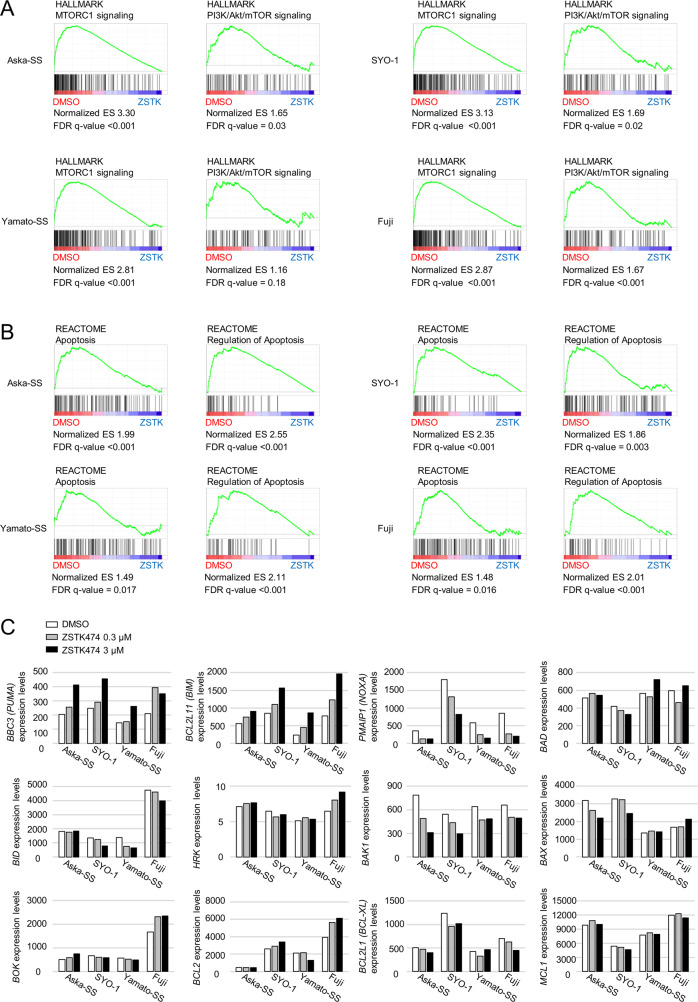
The treatment of synovial sarcoma cells with PI3K inhibitors inhibited PI3K signaling, induced apoptosis and increased the expression of PUMA and BIM in comprehensive gene expression analysis. Aska-SS, SYO-1 Yamato-SS and Fuji cells were treated with the PI3K inhibitor ZSTK474 at 3 μM for 24 h and subjected to microarray analysis. **A**, **B** Representative GSEA plots showing enrichment for the gene signature associated with PI3K signaling (**A**) and apoptosis (**B**) in the indicated cell lines treated with and without ZSTK474. **C** Microarray expression data of BCL-2 family genes in Aska-SS, SYO-1 Yamato-SS and Fuji cells treated with ZSTK474 at the indicated concentrations for 24 h.

To further investigate the effect of PI3K inhibitors on TRS cell lines, we compared the transcriptome of SS cell lines treated with or without ZSTK474. Gene set enrichment analyses (GSEA) demonstrated that SS cells treated with ZSTK474 significantly reduced the signatures of PI3K/Akt/mTOR signaling. Furthermore, analysis revealed that ZSTK474 triggered gene expression changes in the Reactome pathways involved in apoptosis regulation (Fig. 3A and B). Gene ontology (GO) analysis identified GO terms related to cell cycle regulation (GO: 0000278: mitotic cell cycle; GO: 0010564: regulation of cell cycle process) (Fig. S2A). ZSTK474 significantly increased the population of cells in G1 phase of the cell cycle in Aska-SS and SYO-1 cells (Fig. S2B), as well as various carcinoma cell lines, as we demonstrated previously [23, 24]. In addition to the cell cycle, GO terms related to various biological events including cellular response to DNA damage stimulus (GO:0006974), DNA replication (GO:0006260), organelle assembly (GO:0070925), protein localization to organelle (GO:0033365), positive regulation of cell death (GO:0010942) and mitochondrion organization (GO:0007005) were also identified; these processes could all be involved in the strong antitumor effect of ZSTK474 in SS cell lines.

### PUMA and BIM proteins were upregulated after exposure to PI3K inhibitors in TRS cell lines

**Fig. 4:**
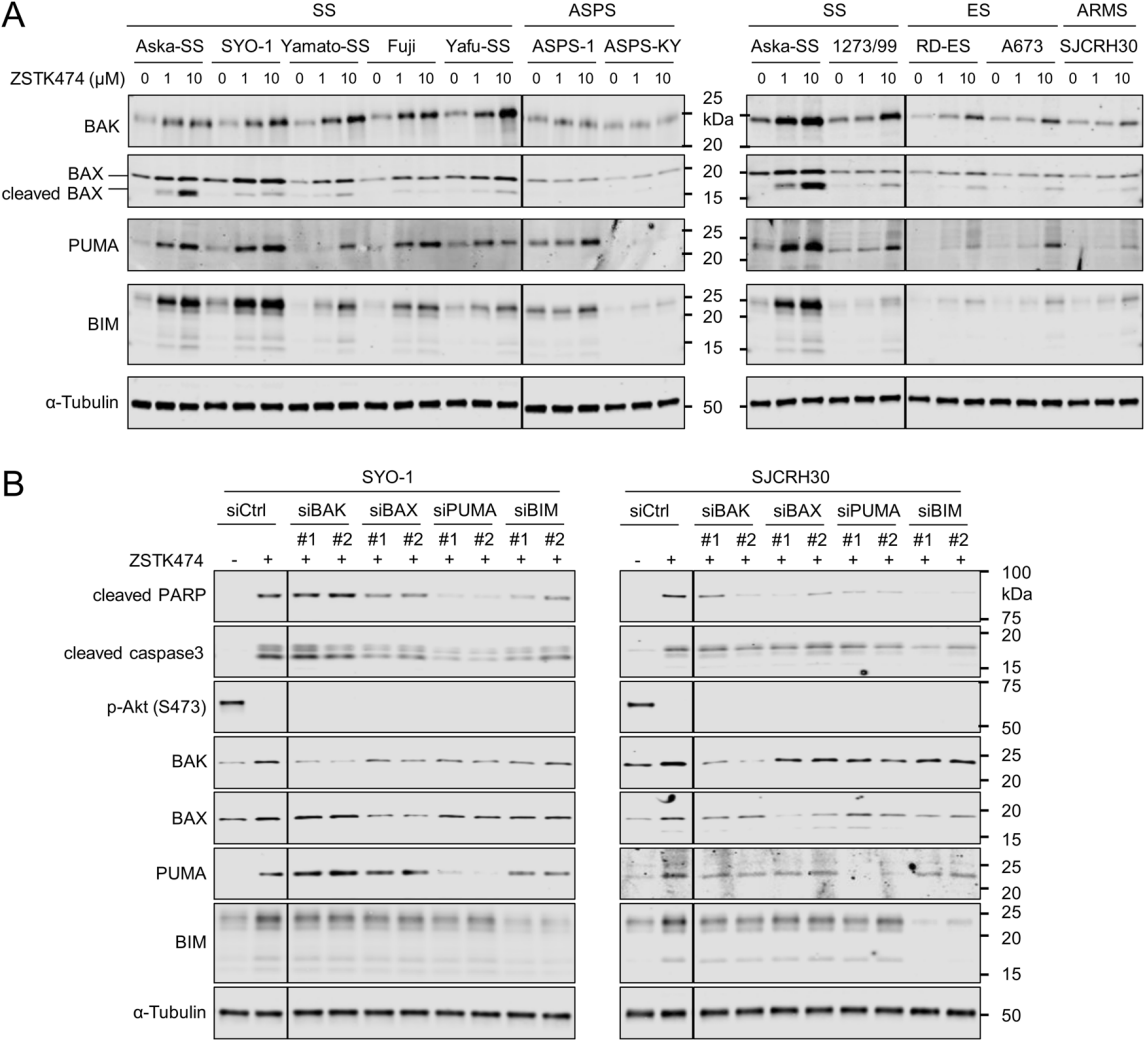
An increase in the expression of BIM and PUMA and the subsequent activation of BAK/BAX contribute to the induction of apoptosis by a PI3K inhibitor. **A** Immunoblot analysis to examine the effect of the PI3K inhibitor ZSTK474 on the expression of proapoptotic BCL-2 family proteins including BAK, BAX, PUMA and BIM in the indicated TRS cell lines including SS, ES, ARMS and ASPS. TRS cells were treated with ZSTK474 at the indicated concentrations for 48 h. **B** Effect of siRNAs specific to BAK, BAX, PUMA and BIM on the induction of apoptosis by ZSTK474. TRS cells transfected with siRNAs were incubated with or without ZSTK474 at 10 μM for 24 h. Lysed samples were immunoblotted to detect BAK, BAX, PUMA, BIM, phosphorylated Akt at Ser473, cleavage of PARP, activation of caspase3 and α-Tubulin. These experiments were performed independently at least two times with similar results.

The BCL-2 family of proteins, which include anti-apoptotic, BH3-only and multi-BH domain-containing proteins, are known to play critical roles in mitochondria membrane permeabilization [25–27]. The genes encoding these proteins are involved in the positive regulation of cell death (GO:0010942) and mitochondrion organization (GO:0007005), as shown by GO analysis (Fig. S2A). In addition, treatment with PI3K inhibitors increased permeability of the mitochondria membrane in SS, ES and ARMS cells (Fig. 2). Thus, we investigated the effect of ZSTK474 treatment on the expression of BCL-2 family members at the mRNA and protein levels in TRS cell lines. Notably, of the genes encoding BH3-only proapoptotic proteins, *BBC3* (PUMA) and *BCL2L11* (BIM) significantly induced in all four SS cell lines upon treatment with ZSTK474 (Fig. 3C). In parallel, the induction of PUMA and BIM were observed at the protein level in SS, and also in ES and ARMS-derived cell lines undergoing apoptosis upon ZSTK474 treatment (Fig. 4A). In contrast, such events were not observed in cell lines derived from ASPS and non-TRS subtypes, all of which did not undergo apoptosis upon ZSTK474 treatment (Fig. 4A and Fig. S1). The expression of multi-BH domain-containing proapoptotic proteins, including BAK and BAX, were detected in all TRS cell lines without drug exposure and their expression levels were slightly upregulated by exposure to ZSTK474 (Fig. 4A). Since it has been reported that BAX is cleaved from a 21 kDa native form to an 18 kDa fragment in response to death stimuli [28, 29], we examined whether treatment with ZSTK474 induces the levels of cleaved BAX. In parallel with the progression of apoptosis, cleaved BAX was observed following ZSTK474 treatment in SS, ES and ARMS but not ASPS (Fig. 4A). With regards to anti-apoptotic BCL-2 family members, the mRNA expression of BCL-2, BCL-XL and MCL-1 was hardly decreased by ZSTK474 in SS cell lines (Fig. 3C, although their protein expression levels were increased, rather than decreased, upon ZSTK474 treatment in SS, ES and ARMS cell lines (Fig. S3)). Taken together, of the BCL2 family proteins examined, the induction of PUMA and BIM is the most relevant event to apoptosis induction by PI3K blockade and is likely to be functionally involved in the progression of apoptosis.

### Induction of FOXO1/3, but not p53, in TRS cell lines undergoing apoptosis triggered by PI3K blockade

It was previously reported that PI3K inhibition induces apoptosis by upregulating forkhead box O1/3 (FOXO1/3), transcription factors downstream of PI3K signaling, and thereby transactivate the expression of PUMA or BIM in hepatocellular carcinoma and colorectal cancer, respectively [30, 31]. Other studies demonstrated that apoptotic stimuli by DNA damaging agents activate p53, leading to apoptosis *via* the transcriptional upregulation of PUMA, NOXA, BID and BAX [32–35]. In this study, to explore the involvement of FOXO1/3 and p53 in the transcriptional upregulation of PUMA and BIM after exposure to PI3K inhibitors, we examined their expression after exposure to a PI3K inhibitor. As a result, the upregulation of FOXO1 and/or FOXO3 was observed in most of the cell lines with upregulated PUMA/BIM expression, but not in ASPS and non-TRS subtypes cell lines which did not undergo apoptosis (Fig. S1 and S4A). In contrast, the induction of p53 protein was not observed in any of the sarcoma cell lines examined, regardless of apoptosis induction (Fig. S4B). These results suggest that the induction of FOXO1 and FOXO3, but not p53, could be involved in the transcriptional activation of PUMA and BIM after PI3K blockade.

### The upregulation of PUMA and BIM contributed to apoptosis induced by a PI3K inhibitor

To determine whether the induction of PUMA and BIM expression and the subsequent activation of BAK/BAX contributed to the induction of apoptosis by PI3K inhibitors, we employed specific siRNAs to knockdown BAK, BAX, PUMA or BIM in TRS cells treated with a PI3K inhibitor. The transfection of siRNA resulted in the partial knockdown of BAK, BAX, PUMA and BIM, and in parallel, impaired the activation of caspase3 and the cleavage of PARP by ZSTK474 treatment in SYO-1 and SJCRH30 cells, except for BAK in SYO-1 cells (Fig. 4B). These results suggest that ZSTK474 induces apoptosis in TRSs including SS, ES and ARMS at least in part through the induction of PUMA and BIM expression and the subsequent activation of BAK/BAX.

### ZSTK474 induced apoptosis and the expression of PUMA and BIM in SS cell lines in vivo

**Fig. 5:**
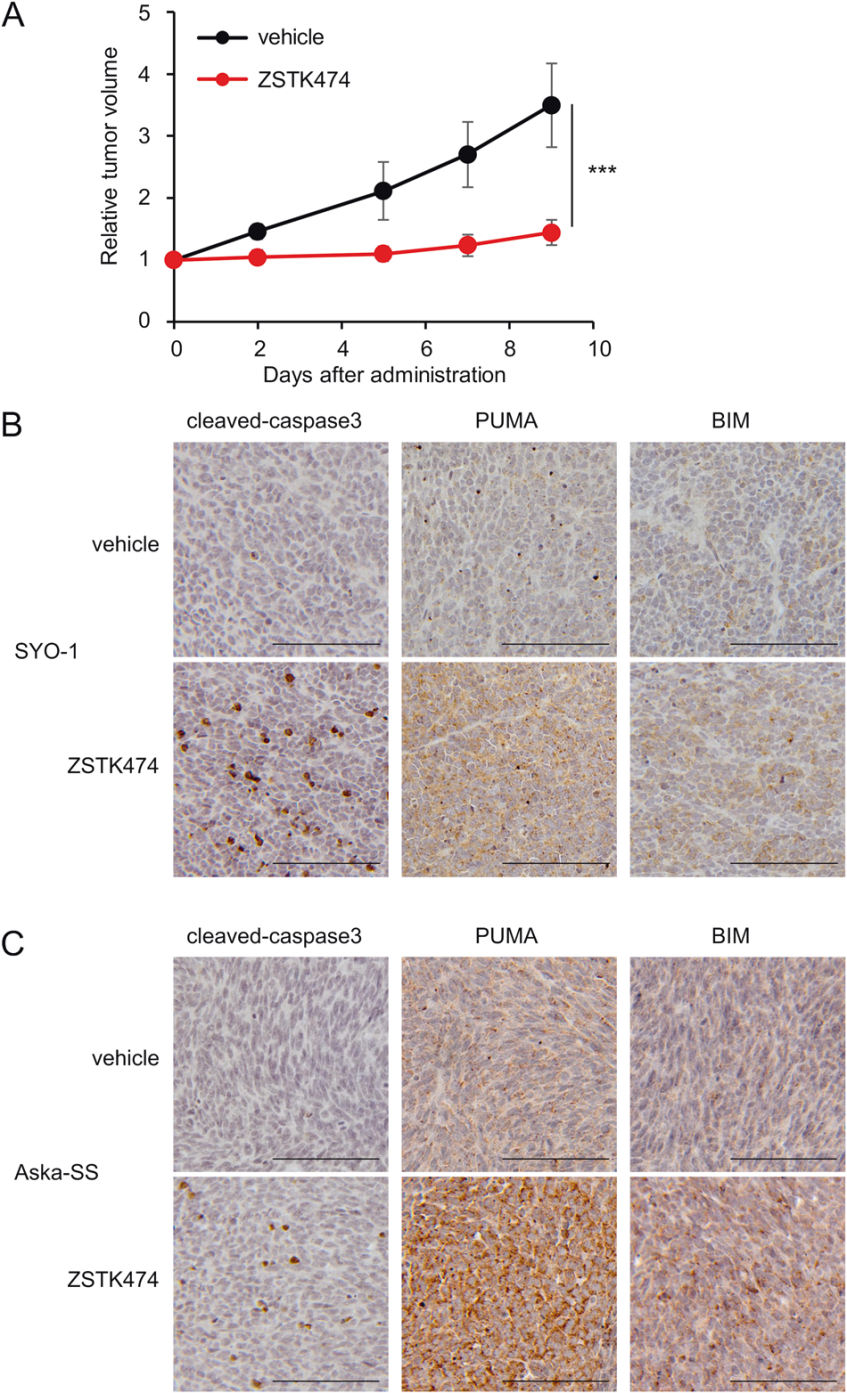
Administration of a PI3K inhibitor induced apoptosis and the expression of PUMA and BIM resulting in a preferable antitumor effect in a SYO-1 and Aska-SS xenograft model. **A** In vivo antitumor activity of the PI3K inhibitor ZSTK474 against Aska-SS xenografts. Data are means ± SD. *n* = 9 for a vehicle group and *n* = 7 for a ZSTK474 treated group. Statistical analysis was performed by Welch’s *t*-test. ***P < 0.001. **B**, **C** The expression levels of cleaved caspase3, PUMA and BIM in SYO-1 (**B**) and Aska-SS (**C**) xenograft tumors from mice treated with or without ZSTK474 at 400 mg/kg once a day for 3 days were analysed by immunohistochemistry. Scale bars, 100 μm.

We previously reported that the administration of ZSTK474 to mice bearing SYO-1 xenografts exhibited a preferable antitumor effect [20]. To examine whether ZSTK474 induced apoptosis and the expression of PUMA and BIM resulting in a preferable antitumor effect in in vivo xenografted tumors, we employed mice bearing Aska-SS xenografts as well as SYO-1. Similar to SYO-1 xenografts, for which we reported high sensitivity in the previous paper [20], the treatment of mice bearing Aska-SS xenografts with ZSTK474 achieved remarkable antitumor effects (Fig. 5A). Immunohistochemistry revealed that the administration of ZSTK474 induced apoptosis, as shown by the increased activation of caspase3 in the xenograft tumors; furthermore, ZSTK474 induced the upregulation of PUMA and BIM (Fig. 5B and C). These results strongly suggest that ZSTK474 induced apoptosis via the induction of PUMA and BIM expression in SYO-1 and Aska-SS tumors in vivo, as well as in vitro.

### PI3K inhibitors induced apoptosis and the expression of PUMA and/or BIM in PDCs of TRSs

**Fig. 6:**
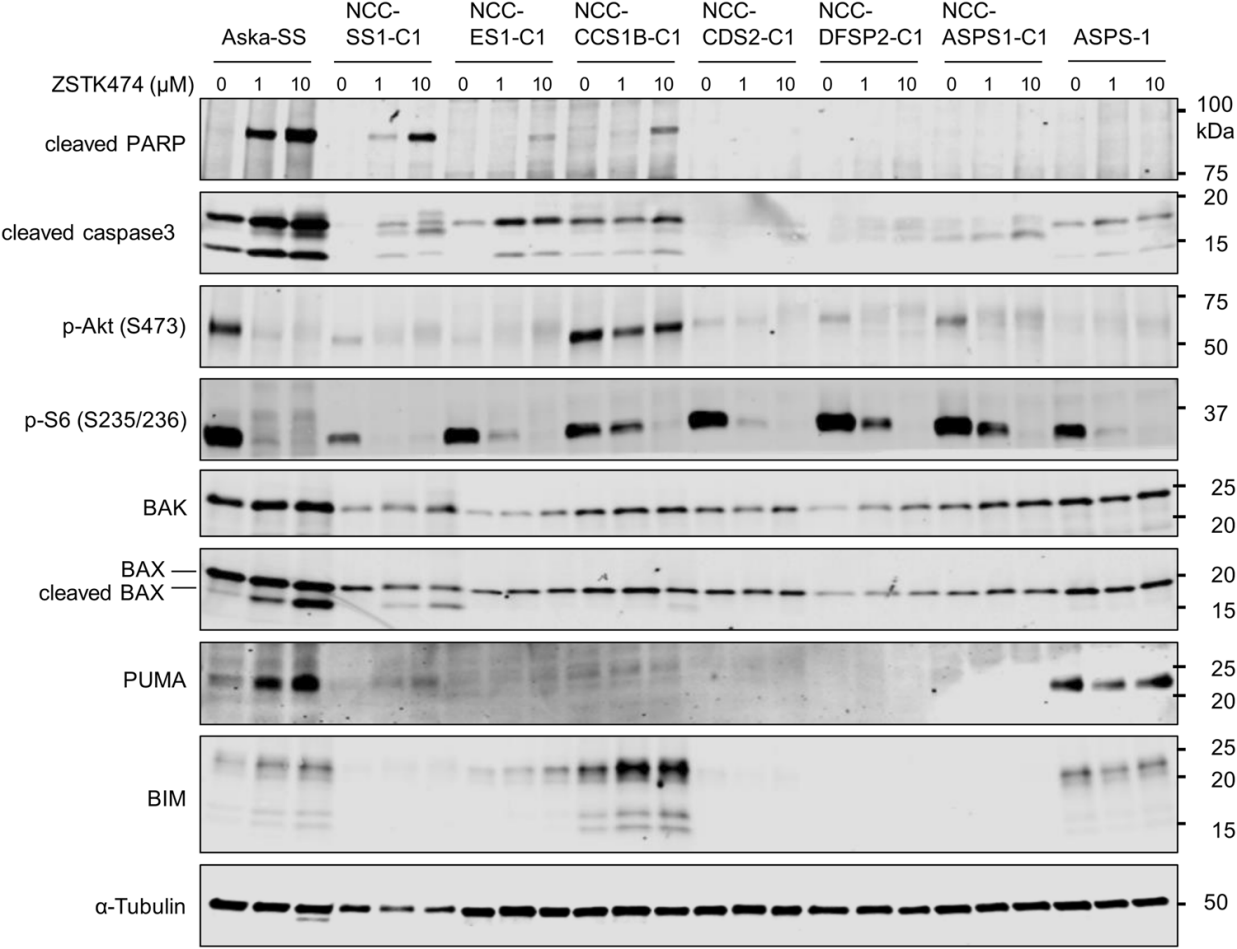
Treatment with a PI3K inhibitor induced the expression of PUMA and/or BIM and the activation of BAK/BAX, thus leading to apoptosis in patient derived cells (PDCs) of TRSs. The effects of the PI3K inhibitor ZSTK474 on PI3K signaling, induction of apoptosis and the expression of proapoptotic BCL-2 family proteins in PDCs of TRSs including SS (NCC-SS1-C1), ES (NCC-ES1-C1), clear cell sarcoma (NCC-CCS1B-C1), CIC-DUX4 sarcoma (NCC-CDS2-C1), dermatofibrosarcoma protuberans (NCC-DFSP2-C1) and ASPS (NCC-ASPS1-C1). PDCs were treated with ZSTK474 at the indicated concentrations for 48 h. Lysed samples were immunoblotted to detect the phosphorylation of Akt (Ser473) and S6 (Ser235/236), cleavage of PARP, activation of caspase3 and the expression of BAK, BAX, PUMA, BIM and α-Tubulin.

To further investigate the effect of PI3K inhibitors in a wide variety of TRS subtypes, we exploited a series of patient-derived tumor cells (PDCs) originally established from surgical tumor specimens of various origins including SS, ES, ASPS, clear cell sarcoma (CCS, harboring the *EWSR1-ATF1* fusion gene), CIC-DUX4 sarcoma (CDS, harboring the *CIC-DUX4* fusion gene) and Dermatofibrosarcoma protuberans (DFSP, harboring the *COL1A1-PDGFB* fusion gene). In accordance to the results obtained from cell lines derived from SS, ES and ASPS in Fig. 1A, ZSTK474 induced apoptosis in PDCs established from SS (NCC-SS1-C1) [36], ES (NCC-ES1-C1) [37] but not in ASPS (NCC-ASPS1-C1) [38]. Furthermore, ZSTK474 also induced apoptosis in PDCs derived from CCS (NCC-CCS1B-C1) [39], but not in those derived from CDS (NCC-CDS2-C1) [40] and DFSP (NCC-DFSP2-C1) [41] (Fig. 6). Consistent with the induction of apoptosis, the expression of activated BAX was detected in NCC-SS1-C1 and NCC-CCS1B-C1 but not NCC-CDS2-C1, NCC-DFSP2-C1 and NCC-ASPS1-C1, although activated BAX was barely detected in NCC-ES1-C1 (Fig. 6). In addition, PUMA and/or BIM were induced by treatment with ZSTK474 in NCC-SS1-C1, NCC-ES1-C1 and NCC-CCS1B-C1 but not NCC-CDS2-C1, NCC-DFSP2-C1 and NCC-ASPS1-C1 (Fig. 6). Similar results were obtained with copanlisib treatment (Fig. S5). Across the sarcoma cell lines and PDCs tested in this study, the expression levels of PUMA and BIM were significantly upregulated by treatment with ZSTK474 in cell lines undergoing apoptosis, while PUMA and BIM expression was not induced in cell lines not undergoing apoptosis (Fig. S6). These results suggest that PI3K inhibitors selectively induce apoptosis in SS, ES, ARMS and CCS but not CDS, DFSP and ASPS due to the induction of PUMA and BIM expression and thereby the activation of BAK/BAX.

## Discussion

In this study, we aimed to determine whether and how PI3K inhibitors induced apoptosis in different TRS subtypes using an expanded sarcoma cell panel including cell lines and PDCs. The major finding of this study is that PI3K inhibitors induced apoptosis in TRSs especially in SS, ES, ARMS, and CCS, but not ASPS. We also found that PI3K inhibitors increased mitochondrial outer membrane permeability, the activation of BAK/BAX, the upregulation of PUMA, BIM, FOXO1 and FOXO3 expression. Thus, we concluded that PI3K inhibitors selectively induced apoptosis in TRSs such as SS, ES, ARMS, and CCS in a manner that was mediated by increased mitochondrial outer membrane permeability via the upregulation of PUMA and BIM expression probably due to transcriptional activation by FOXO1 and FOXO3.

All of the 12 cell lines from SS (7), ES (3), ARMS (1) and CCS (1) underwent apoptosis, while cell lines from ASPS (3), DFSP (1) and CDS (1) did not. Notably, the induction of apoptosis after exposure to PI3K inhibitors correlated quite well with the induction of PUMA and BIM, whereas the substantial inhibition of PI3K-downstream signaling, as demonstrated by the downregulation of phosphorylated Akt, was observed in cell lines not undergoing apoptosis, as well as in those undergoing apoptosis. In other words, PI3K signaling seems to play an important role in maintaining cell survival by repressing the expression of PUMA and BIM in cell lines including SS and ES, which both exhibited susceptibility to PI3K blockade.

The involvement of BCL-2 family proteins in the progression of apoptosis triggered by other molecular target drugs has been reported previously. For example, EGFR inhibitors and BRAF inhibitors induce apoptosis by increasing the expression of BH3 only proapoptotic proteins such as BIM, PUMA and NOXA, and/or by decreasing expression of anti-apoptotic proteins such as BCL-2 and MCL-1 in cells derived from EGFR-mutant non-small cell lung cancers and BRAF-mutant melanomas, respectively [42–44]. In osteosarcoma, dinaciclib, a pan-cyclin-dependent kinase inhibitor, induced apoptosis by suppressing the expression of MCL-1 and BCL-XL and by inducing the expression of BIM [45]. In the present study, PI3K inhibitors upregulated proapoptotic BCL family members including PUMA and BIM both at the mRNA and protein levels, whereas neither of the anti-apoptotic BCL family members including BCL-2, BCL-XL and MCL-1 were not downregulated in TRS-derived cell lines such as SS, ES and ARMS. The upregulation of FOXO1 and/or FOXO3 in cells undergoing apoptosis after blockade of the PI3K pathway suggested the involvement of FOXO1/3 in the induction of PUMA and BIM, as previously reported in cell lines derived from hepatocellular carcinoma and colorectal cancers [30, 31]. FOXO transcription factors are known to be downstream factors of the PI3K/Akt signaling pathway and their expression can be degraded by the ubiquitin-proteasome pathway via phosphorylation by Akt [46]. Therefore, the inhibition of PI3K is likely to upregulate FOXO1/3 and thereby transactivate the expression of PUMA and BIM. In contrast, Hata et al. reported that BIM protein levels are regulated by MEK/ERK signaling while the expression of PUMA is regulated by PI3K signaling in cancers driven by oncogenic driver mutations on tyrosine kinase, thus resulting in the constitutive activation of downstream kinase signaling pathways including the MEK/ERK and PI3K signaling pathways [44]. In our present study, the phosphorylation level of MEK/ERK remained unchanged in TRS cell lines undergoing apoptosis after exposure to ZSTK474 (Fig. 1A), thus suggesting that MEK/ERK signaling does not seem to be involved in the upregulation of BIM.

In the present study, it was unclear as to why SS, ES, ARMS and CCS, but not ASPS, underwent apoptosis upon PI3K blockade. One possible explanation for this difference is that it arises from the preference of upstream receptor tyrosine kinase (RTK) dependence. Reportedly, the proliferation and survival of SS, ES, ARMS and CCS cells, which underwent apoptosis upon PI3K blockade in this study, are dependent on insulin like growth factor 1 receptor (IGF1R) signaling due to the induction of IGF1, IGF2 and IGF1R expression by the respective fusion genes such as *SS18-SSX*, *EWSR1-FLI1*, *PAX3-FOXO1* and *EWSR1-ATF1* [5, 47–49]. In contrast, ASPS cells did not undergo apoptosis and were shown to be preferentially dependent on MET, vascular endothelial growth factor (VEGF) and platelet derived growth factor (PDGF) instead of IGF1R [50]. In general, RTK-downstream signaling is dependent on two major pathways, the PI3K/Akt- and MEK/ERK pathways; of these, IGF1R predominantly utilizes the PI3K/Akt pathway whereas MET, VEGF and PDGF are dependent on both pathways [51, 52]. This may be a reason why cell survival in SS, ES, ARMS and CCS, but not ASPS, was highly dependent on PI3K. Indeed, multi-targeted RTK inhibitors (TKIs) such as sunitinib, VEGFR inhibitors such as pazopanib and bevacizumab, and MET inhibitors such as crizotinib, have been reported to be effective for ASPS in some clinical trials [50, 53, 54].

In conclusion, PI3K inhibitors selectively induced apoptosis in SS, ES, ARMS, and CCS, but not in ASPS among the TRSs examined so far; this effect occurred via the upregulation of PUMA and BIM and the subsequent increased permeability of the mitochondrial membrane; however, the precise regulatory mechanisms underlying the selective induction of apoptosis in cell lines derived from specific TRS subtypes including SS and ES remains to be elucidated. However, our present findings provide us with a promising therapeutic modality that would satisfy unmet needs for patients with such TRS subtypes.

## Materials and methods

### Drugs

ZSTK474 was provided by OHARA Pharmaceutical Co., Ltd. (Tokyo, Japan). Copanlisib was purchased from Selleck Chemicals (Houston, TX) and Z-VAD-FMK was purchased from Adipogen Life Sciences (Liestal, Switzerland). These compounds were dissolved in dimethyl sulfoxide.

### Cell lines and patient derived cells (PDCs)

The cell lines and PDCs used in this study are listed in Table S1 and S2, respectively [10, 55–68]. All cell lines were maintained in RPMI 1640 supplemented with 5% (v/v) fetal bovine serum and 1 μg/mL of kanamycin at 37 °C in humid air containing 5% CO_2_. All PDCs used in this study were established from resected tumor specimens at National Cancer Center Hospital/Research Institute, Tokyo, Japan. PDCs were maintained in DMEM or DMEM/F12 supplemented with 10% (v/v) fetal bovine serum and 1 μg/mL of kanamycin at 37 °C in humid air containing 5% CO_2_.

### Immunoblotting

Immunoblot assays were carried out on cell extracts using the primary antibodies listed in Table S3. Visualization and quantification of the bound antibody was carried out using an anti-rabbit or anti-mouse immunoglobulin secondary antibody labeled with Alexa Fluor Plus 680 or 800 (Thermo Fisher Scientific, Waltham, MA) and the Odyssey Infrared Imaging System (LI-COR Biosciences, Lincoln, NE).

### JC-1 staining

Changes in mitochondrial membrane potential were assessed by JC-1 staining (PK-CA707-70014, PromoCell, Heidelberg, Germany) [22]. Cells were incubated in complete medium with JC-1 at 1.7 μM and verapamil at 50 μM for 30 min at 37 °C. The stained cells were analyzed with BD FACSMelody (BD Biosciences, Franklin Lakes, NJ) and FlowJo software (BD Biosciences).

### Cell cycle analysis

Cell cycle analysis was performed by flow cytometry using the Vybrant DyeCycle Violet (Thermo Fisher Scientific). Cells were incubated in 1 ml of complete medium with 1 μL of Vybrant DyeCycle Violet for 30 min at 37 °C. The stained cells were analyzed with BD FACSmelody (BD Biosciences) and FlowJo software (BD Biosciences).

### RNA interference

siRNAs specific to BAK (Hss184086, Hss184087), BAX (s1888, s1889), PUMA (s25840, s25841), BIM (s195011, s195012) and the negative control siRNA (4390843) were purchased from Thermo Fisher Scientific. Cells were plated on 6-well plates and transfected with 44 nmol/L of siRNAs using RNAiMAX (Thermo Fisher Scientific) according to the manufacturer’s instructions. The transfected cells were then incubated with or without drugs for 24 h and lysed with lysis buffer for immunoblotting [69].

### Mouse xenograft model

Female BALB/c nude mice (The Jackson Laboratory Japan, Kanagawa, Japan) were inoculated subcutaneously (into the back) with Aska-SS cells or SYO-1 cells. When tumors reached 100–300 mm^3^ in volume, the mice were randomized into a vehicle control group and a ZSTK474-treated group. Oral administration was performed once a day with ZSTK474 (400 mg⁄kg suspended in 5% hydroxypropylmethylcellulose in water) except for days 5 and 6 from the day of first administration (day 0). Tumor volume (TV) was monitored three times a week by measurement of the length (L) and width (W) of the subcutaneous tumor mass using calipers; TV was calculated as TV = (L × W^2^)/2. The sample size (*n* = 7 to 9) was based on the results of preliminary experiments. The investigators were not blinded during evaluation in in vivo experiments. The experimental protocol was approved by the Institutional Animal Experimental Committee at Japanese Foundation for Cancer Research.

### Immunohistochemistry

Xenograft tumors were fixed in 10% Formalin Neutral Buffer, embedded in paraffin and sectioned at 4 μm. The sections were deparaffinized in xylene and taken through a series of graded alcohols to water. Endogenous peroxidase activity was quenched using 3% hydrogen peroxide and antigen retrieval was performed by heating at 95°C for 40 min in Target Retrieval Solution Citrate (PH6.0, Dako, Glostrup, Denmark). After blocking with 10% goat serum, the sections were incubated with a primary antibody (Table S3) at 4 °C overnight. Following incubation with HRP-conjugated secondary antibody for 1 h at room temperature, the sections were stained with diaminobenzidine and counterstained with hematoxylin.

### Microarray analysis

Total RNA from TRS cells was extracted by the RNeasy kit (Qiagen, Hilden, Germany). Microarray analysis was performed using the GeneChip Human Genome U133 Plus 2.0 Array (Thermo Fisher Scientific) according to the manufacturer’s instructions. GeneSpring software v.14.9.1 (Agilent, Santa Clara, CA) was used for quantile normalization and data processing. Gene ontology (GO) analysis was performed using Metascape. Gene set enrichment analysis (GSEA) was performed using GSEA v4.1.0 software. PI3K signaling and apoptosis signatures were downloaded from MSigDB: Hallmark mTORC1 signaling, Hallmark PI3K/Akt/mTOR signaling, Reactome Apoptosis and Reactome Regulation of apoptosis.

### Statistics

The significance of differences between groups was determined with Excel and EZR software using one-way analysis of variance (ANOVA) with post hoc Dunnett’s test, one-way repeated measures ANOVA with post hoc Dunnett’s test and unpaired two-tailed Welch’s *t*-test.

## Supplementary information

supplemental Figures

supplemental Table 1

supplemental Table 2

supplemental Table 3

Original Data File

aj-checklist

## Data Availability

The datasets generated or analyzed during the current study are included either in this article or in the supplemental materials files. The microarray data discussed in the current study are available in the Gene Expression Omnibus and the data can be accessed by the accession number GSE221131.
