# Supplementary material for: Subtype-selective induction of apoptosis in translocation-related sarcoma cells induced by PUMA and BIM upon treatment with pan-PI3K inhibitors
Source: Cell Death Dis. 2023 Feb 27;14(2):169. doi: 10.1038/s41419-023-05690-7 (PMC9971170; doi:10.1038/s41419-023-05690-7)
Supplement: Supplementary file 1 — supplemental Figures [file 41419_2023_5690_MOESM1_ESM.docx]

**
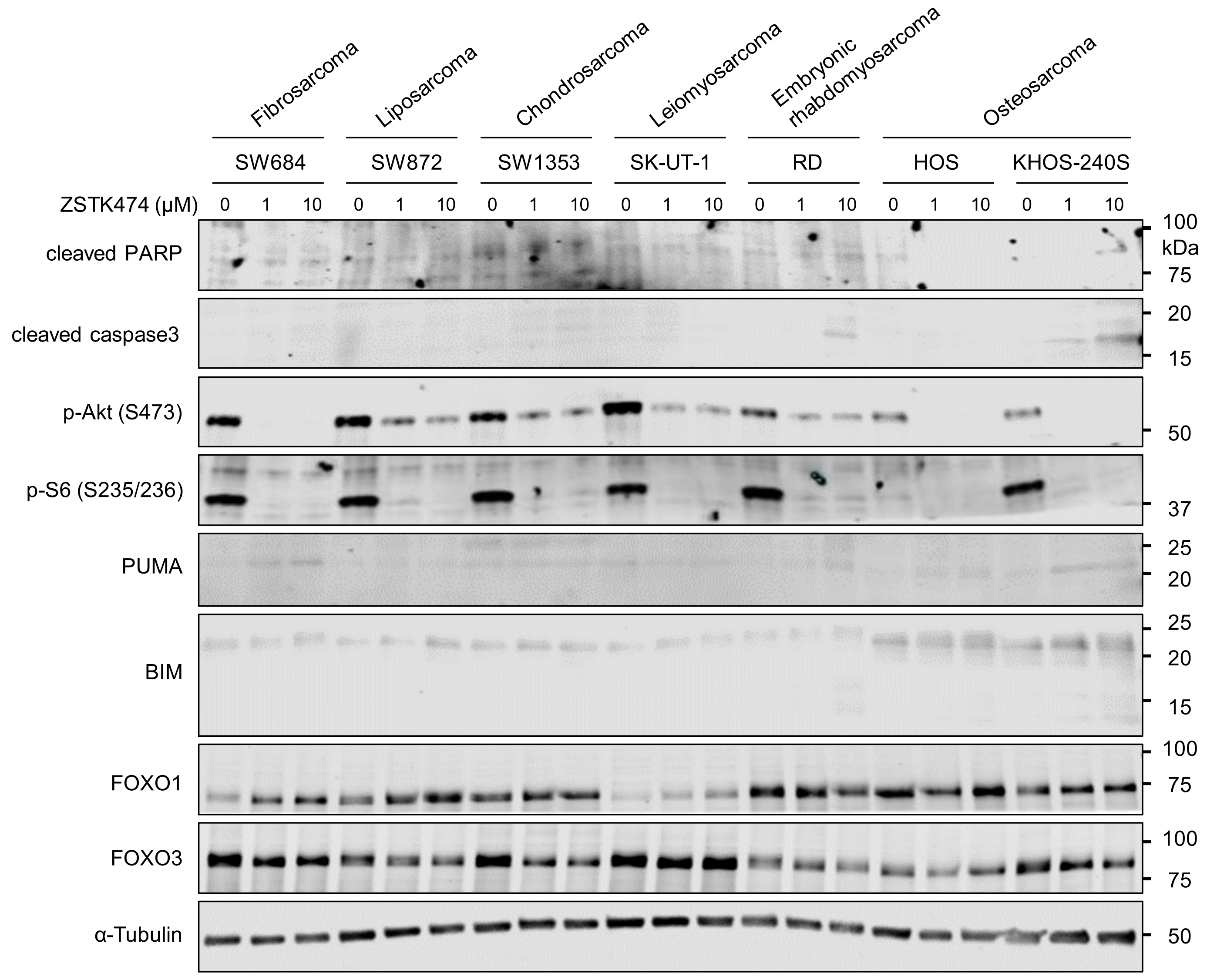
**

**Supplementary Figure 1. Treatment with a PI3K inhibitor failed to induce apoptosis and the expression of PUMA BIM and FOXO1/3 in non-TRS cell lines.**

Effect of the PI3K inhibitor ZSTK474 on PI3K signaling, apoptosis and the expression of BH3 only proteins in non-TRS cell lines including fibrosarcoma (SW684), liposarcoma (SW872) chondrosarcoma (SW1353), leiomyosarcoma (SK-UT-1), embryonic rhabdomyosarcoma (RD) and osteosarcoma (HOS and KHOS-240S). Cells were treated with ZSTK474 at the indicated concentrations for 48 hours. Lysed samples were immunoblotted to detect the phosphorylation of Akt (Ser473) and S6 (Ser235/236), cleavage of PARP, activation of caspase3 and expression of PUMA, BIM, FOXO1, FOXO3 and α-Tubulin. These experiments were performed independently at least two times with similar results.

**
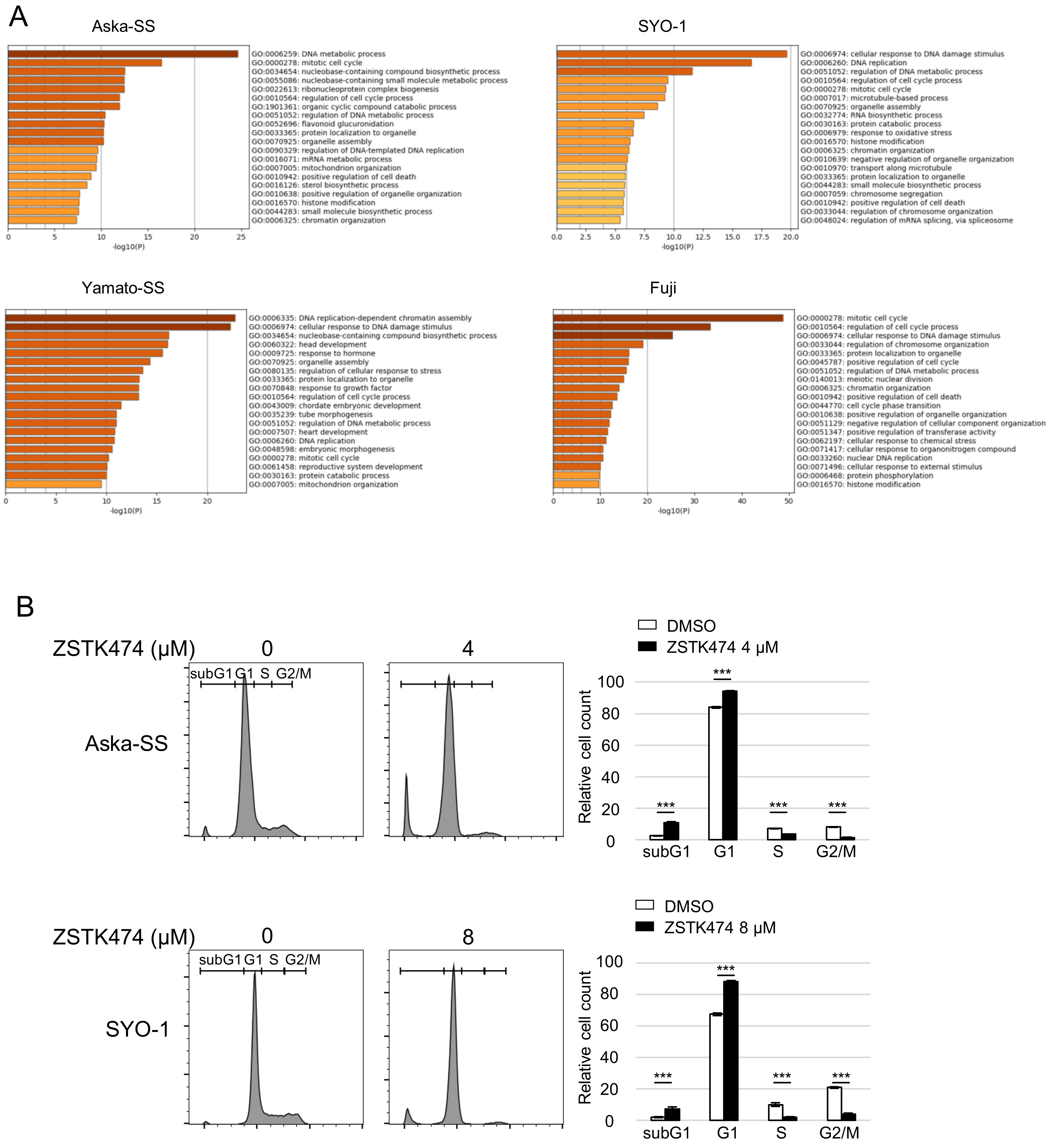
Supplementary Figure 2. The treatment of synovial sarcoma cells with PI3K inhibitors induced G1 arrest in the cell cycle.**

**A** Aska-SS, SYO-1 Yamato-SS and Fuji cells were treated with a PI3K inhibitor ZSTK474 at 3 μM for 24 hours and subjected to microarray analysis. GO analysis of the differentially expressed genes between SS cells treated with and without ZSTK474 was performed. The top 20 enriched GO terms for biological processes were ranked by p-values. **B** Cell cycle analysis by flow cytometry of Aska-SS and SYO-1 treated with ZSTK474 at the indicated concentrations for 48 hours. Representative histograms and summary of the frequencies of indicated cell cycle phases. Data are means ±SD. These experiments were performed in triplicate and independently at least two times with similar results. Statistical analyses were performed by Student's t test (B). ***, P<0.001.

**
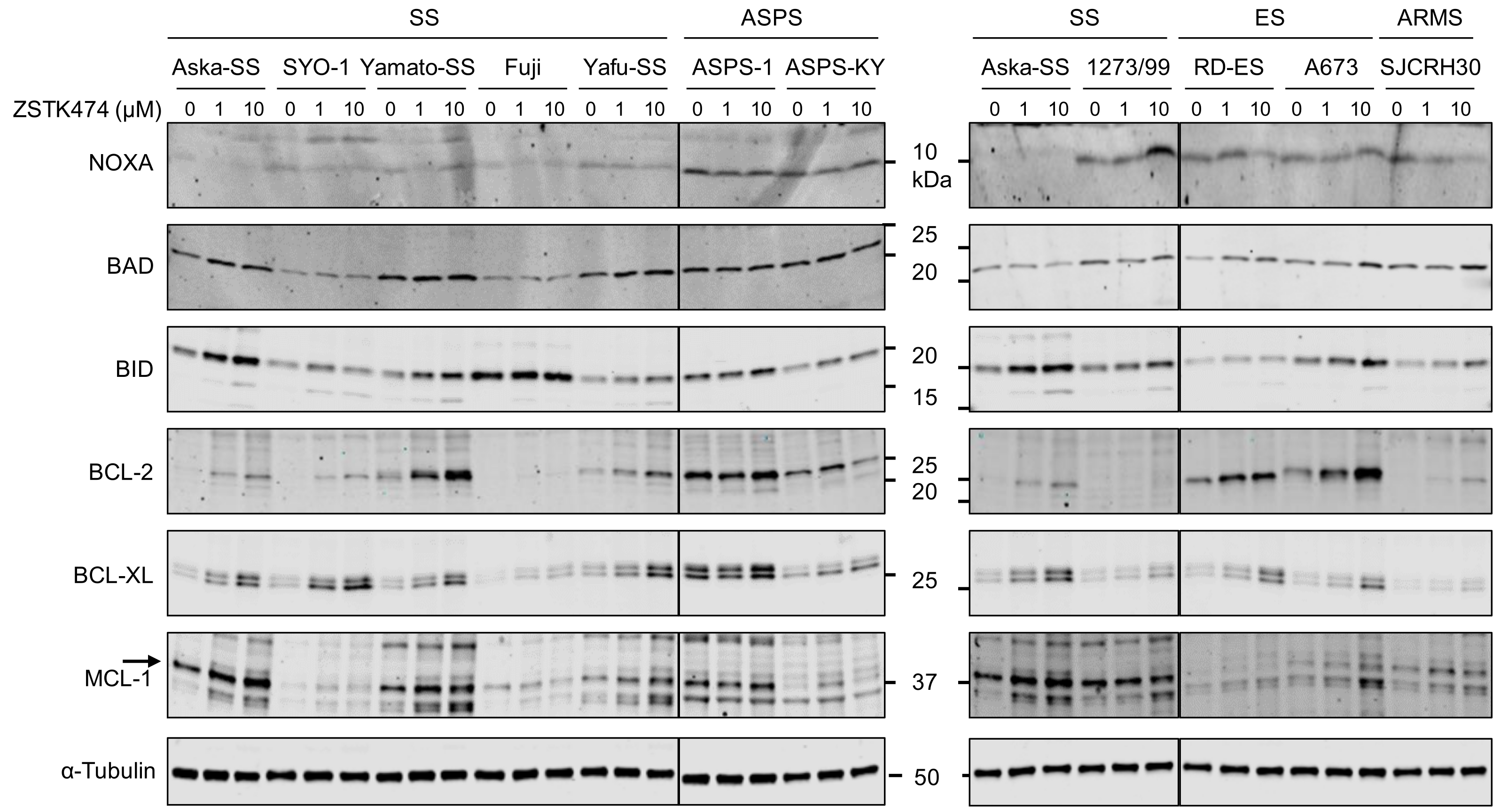
Supplementary Figure 3. Expression of BCL-2 family proteins in translocation-related sarcoma (TRS) cell lines upon treatment with a PI3K inhibitor.**

Immunoblot analysis to examine the effect of the PI3K inhibitor ZSTK474 on the expression of BCL-2 family proteins including NOXA, BAD, BID, BCL-2, BCL-XL and MCL-1 in TRS cell lines including synovial sarcoma (SS), Ewing sarcoma (ES), alveolar rhabdomyosarcoma (ARMS) and alveolar soft part sarcoma (ASPS). TRS cells were treated with ZSTK474 at the indicated concentrations for 48 hours. These experiments were performed independently at least two times with similar results.

**
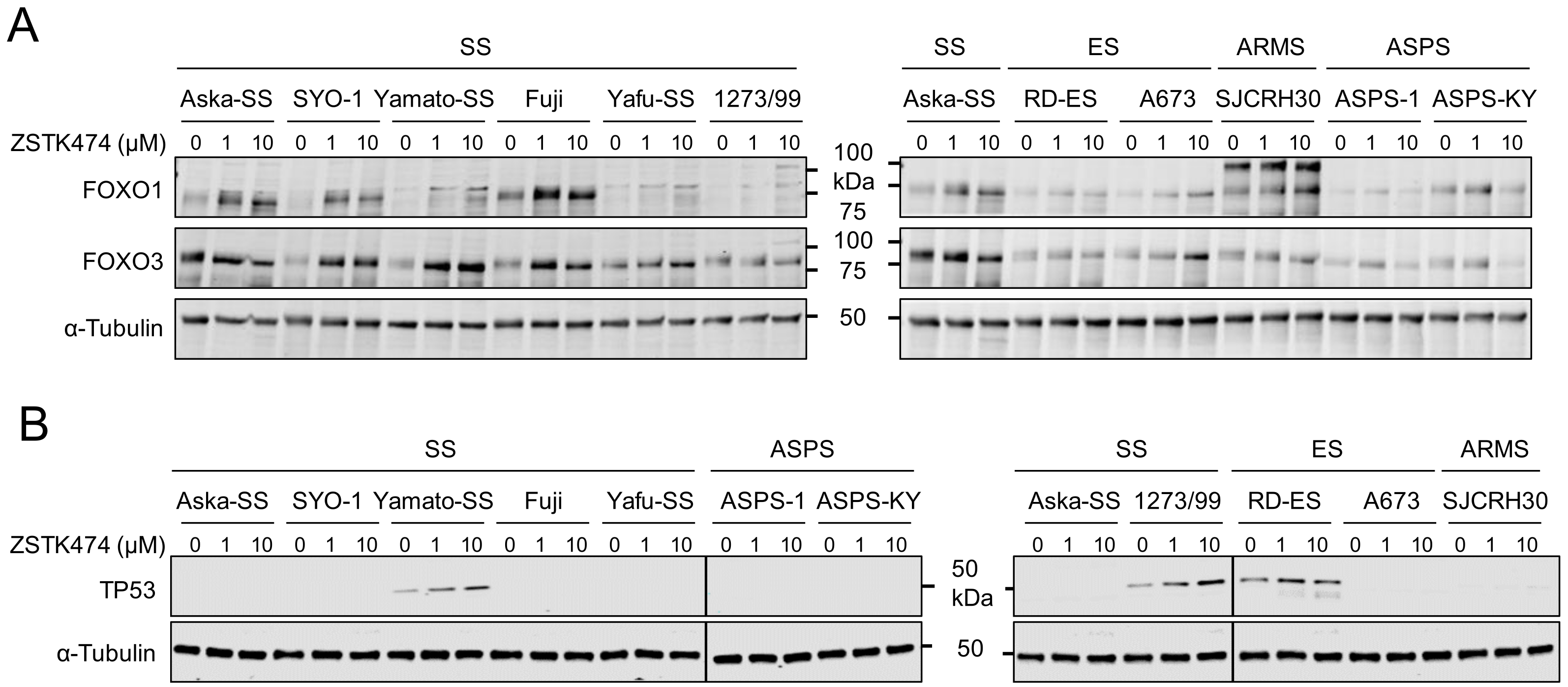
Supplementary Figure 4. Treatment with a PI3K inhibitor induced the expression of FOXO1 and/or FOXO3 but not TP53 in TRS cell lines.**

**A, B** Immunoblot analysis to examine the effect of the PI3K inhibitor ZSTK474 on the expression of FOXO1, FOXO3 (**A**) and TP53 (**B**) in TRS cell lines including synovial sarcoma (SS), Ewing sarcoma (ES), alveolar rhabdomyosarcoma (ARMS) and alveolar soft part sarcoma (ASPS). TRS cells were treated with ZSTK474 at the indicated concentrations for 48 hours.

**
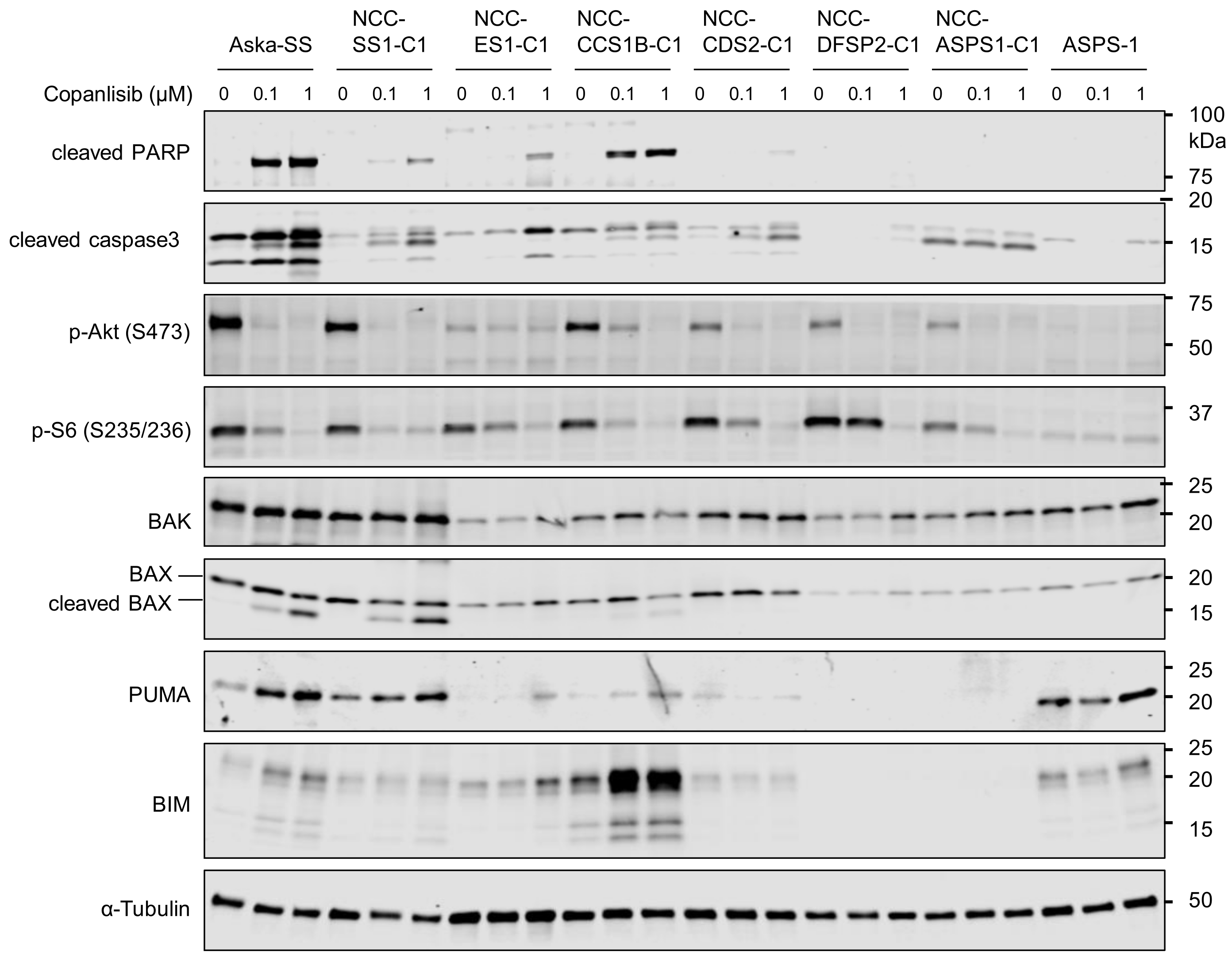
Supplementary Figure 5. Treatment with a PI3K inhibitor induced the expression of PUMA and BIM and the activation of BAK/BAX, thus leading to apoptosis in patient derived cells (PDCs) of TRSs.**

Effect of the PI3K inhibitor copanlisib on PI3K signaling, apoptosis and the expression of proapoptotic BCL-2 family proteins in PDCs of TRSs including SS (NCC-SS1-C1), ES (NCC-ES1-C1), clear cell sarcoma (NCC-CCS1B-C1), CIC-DUX4 sarcoma (NCC-CDS2-C1), dermatofibrosarcoma protuberans (NCC-DFSP2-C1) and ASPS (NCC-ASPS1-C1). TRS cells were treated with copanlisib at the indicated concentrations for 48 hours. Lysed samples were immunoblotted to detect phosphorylation of Akt (Ser473) and S6 (Ser235/236), cleavage of PARP, activation of caspase3 and expression of BAK, BAX, PUMA, BIM and α-Tubulin.

**
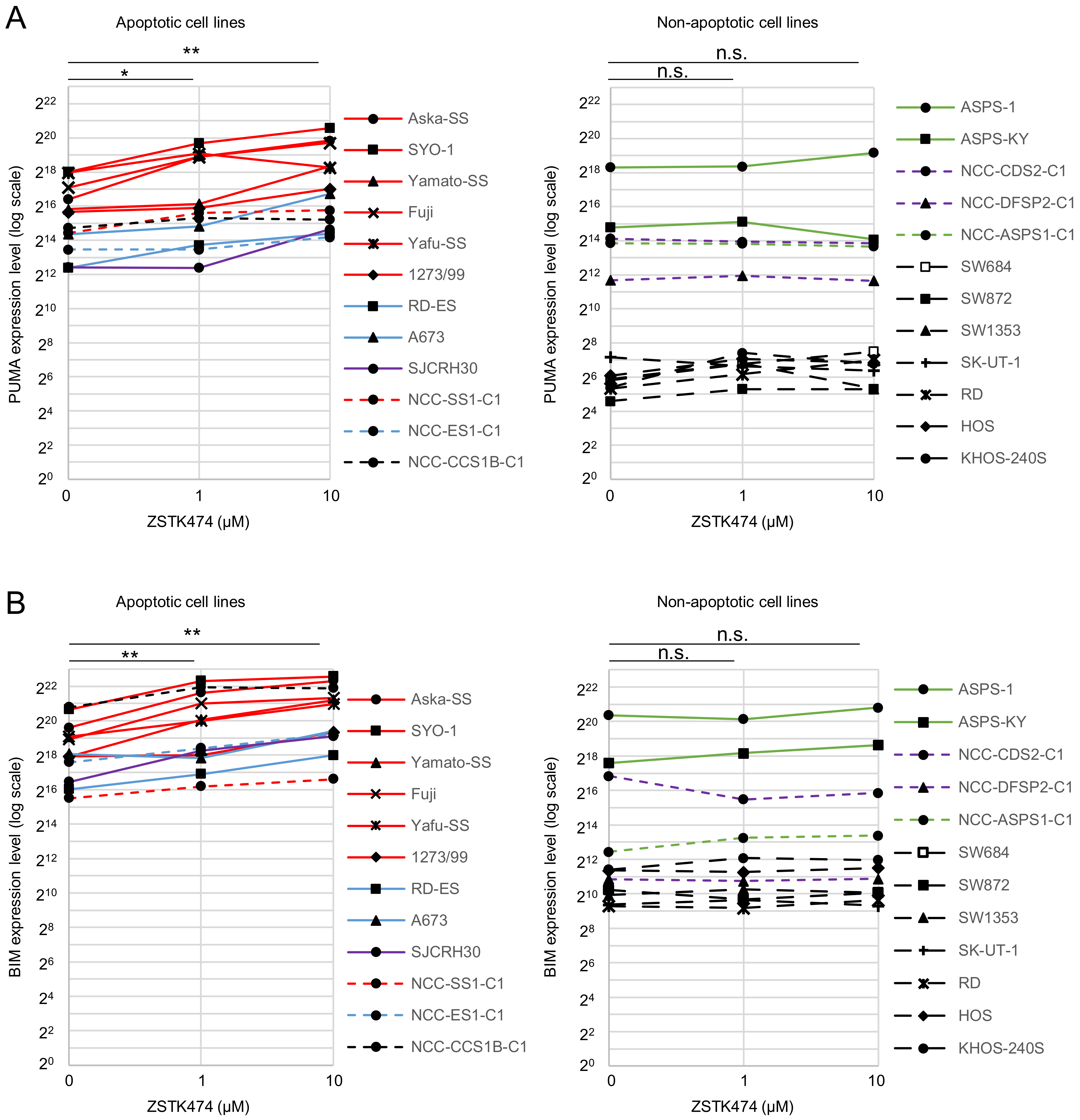
Supplementary Figure 6. Treatment with a PI3K inhibitor induced the expression of PUMA and BIM in apoptotic cells but not non-apoptotic cells.**

**A, B** The expression levels of PUMA (**A**) and BIM (**B**) in apoptotic (left panel) and non-apoptotic cell lines (right panel) treated with a PI3K inhibitor at the indicated concentrations for 48 hours. The expression levels were quantified by immunoblot analyses shown in **Fig. 4A**, **Fig. 6** and **Fig. S1**. Statistical analyses were performed by Dunnett’s test. *, P < 0.05; **, P < 0.01; ***, P<0.001.
